# Machine learning-based integration of systemic immune-inflammation and nutritional signatures for predicting disease-free survival in upper tract urothelial carcinoma: a multicenter study

**DOI:** 10.3389/fimmu.2026.1759547

**Published:** 2026-04-22

**Authors:** Xiang Peng, Bangxin Xiao, Zhanpeng Yuan, Yingjie Xv, Mingzhao Xiao, Wei Shi

**Affiliations:** 1 Department of Urology, The First Affiliated Hospital of Chongqing Medical University, Chongqing, China; 2 Center for Reproductive Medicine, Women and Children’s Hospital of Chongqing Medical University, Chongqing Health Center for Women and Children, Chongqing, China

**Keywords:** machine learning, prognosis, random survival forest, systemic immune-inflammation index, upper tract urothelial carcinoma

## Abstract

**Background:**

Current staging for Upper Tract Urothelial Carcinoma (UTUC) fails to capture host biological heterogeneity. We aimed to develop and validate a machine - learning based prognostic signature integrating systemic immune - inflammatory and nutritional markers to enhance UTUC risk stratification.

**Methods:**

A total of 606 UTUC patients from four centers were divided into a training set (n = 263), an internal validation set (n = 114), and two external validation sets (n = 113, n = 116). Thirteen preoperative hematological markers were screened using LASSO - Cox regression. A Random Survival Forest (RSF) algorithm was utilized to construct a prognostic “ML Score”, and SHAP analysis visualized the nonlinear relationships. A composite nomogram integrating the ML Score with clinical factors (age, grade, pT stage) was developed and comprehensively evaluated.

**Results:**

Seven principal predictors were identified: RDW, PLT, NEUT, NLR, SIRI, SII, and AISI. SHAP analysis revealed distinct nonlinear threshold effects of RDW and PLT on mortality risk. The ML Score served as an independent prognostic indicator, successfully identifying patients with significantly poorer disease - free survival (DFS) across all four cohorts (P < 0.01). The integrated nomogram demonstrated outstanding predictive accuracy, with a C - index of 0.762 in the training set and maintaining robust performance in all validation cohorts. Decision curve analysis confirmed its superior clinical net benefit.

**Conclusion:**

We developed and validated a robust ML Score that reflects the host’s systemic immune - inflammatory and nutritional status. It offers substantial incremental prognostic value and serves as an accurate, non - invasive tool for personalized risk assessment in UTUC.

## 1 Introduction

Upper Tract Urothelial Carcinoma (UTUC), a malignant tumor originating from the renal pelvis or ureteral urothelium, accounts for 5 – 10% of all urothelial carcinomas (1). Despite its relatively low incidence, this malignancy is characterized by highly aggressive biological behavior (2). Radical nephroureterectomy (RNU) with bladder cuff excision remains the gold - standard treatment for high - risk UTUC (1, 3, 4). However, postoperative outcomes exhibit substantial variability. Approximately 22% to 47% of patients encounter intravesical recurrence within the follow - up period, and the 5 - year cancer - specific survival (CSS) rates differ notably across diverse risk groups (1, 5, 6). Current clinical decision - making predominantly depends on the American Joint Committee on Cancer (AJCC) TNM staging system and the WHO pathological grading. Nevertheless, the unique anatomy of the upper urinary tract often precludes accurate preoperative assessment of tumor invasion depth (T stage) via biopsy, leading to postoperative pathological upstaging in up to about 40% of patients (7–10). More critically, current anatomical staging systems depend solely on tumor burden, neglecting the “host factor”—specifically, the heterogeneity of the host’s systemic immune and nutritional response to malignancy (11). Clinically, patients with the same staging often display significantly different prognoses, emphasizing an urgent requirement for new non-invasive biomarkers that mirror “host-tumor interaction” to supplement existing systems, thus facilitating more accurate preoperative risk stratification and personalized treatment decisions (12).

Mounting evidence indicates that cancer-related inflammation (CRI) is a hallmark of malignancy (13). The systemic inflammatory response not only reflects the immune status of the host but also actively facilitates the remodeling of the tumor microenvironment (TME), thereby promoting proliferation, invasion, and distant metastasis (14). Peripheral blood hematological parameters, acting as a “window” into the host’s systemic immune-inflammatory state, harbor rich prognostic information. Indeed, recent evidence has underscored that the integration of systemic diseases and serum biomarkers—particularly those reflecting inflammation and nutrition—plays a vital role in capturing the host’s systemic health status (15). Specifically, neutrophils secrete vascular endothelial growth factor (VEGF) and matrix metalloproteinases (MMPs) to stimulate angiogenesis and capture circulating tumor cells (CTCs) via neutrophil extracellular traps (NETs), thereby promoting metastatic colonization (16–18). Platelets hinder natural killer (NK) cells by acquiring major histocompatibility complex (MHC) class I and forming a protective “mantle” around tumor cells to facilitate immune evasion (19). In contrast, lymphocytes (especially CD8+ T cells) play a crucial role in the anti - tumor immune response, and their depletion often indicates impaired immune surveillance (20). Furthermore, the red blood cell distribution width (RDW), which was traditionally used to measure the heterogeneity of erythrocyte volume, has been re - defined as a novel biomarker for inflammation and nutritional status. Chronic inflammatory cytokines (e.g., IL - 6, TNF - α) suppress the activity of erythropoietin (EPO) and interfere with iron metabolism, resulting in an increase in RDW. Furthermore, a high RDW is significantly associated with oxidative stress and cancer cachexia (21–24). Consequently, the integration of these multidimensional biomarkers, which mirror inflammation, immunity, coagulation, and nutrition, provides a unique biological vantage point for prognosticating the outcome of UTUC.

Although individual composite indices, including the neutrophil - to - lymphocyte ratio (NLR) and systemic immune - inflammation index (SII), have been investigated in UTUC (25, 26), previous studies exhibit two notable limitations. Firstly, they depend on one - dimensional metrics that are unable to comprehensively depict the entire systemic immune - inflammatory landscape. Secondly, they primarily employ traditional Cox proportional hazards regression models. Conventional linear statistical methods assume a linear association between biomarkers and risk (Linearity Assumption). Nevertheless, biological processes are intrinsically complex and nonlinear (27). For example, certain indicators may only notably elevate the mortality risk after surpassing specific thresholds, a nonlinear effect frequently neglected by traditional models. Machine learning (ML), especially the Random Survival Forest (RSF) algorithm, presents a potent solution. As an ensemble learning approach, RSF is not restricted by the proportional hazards assumption, facilitating the automatic processing of high - dimensional data, the identification of crucial features, and the accurate modeling of nonlinear relationships and intricate interactions (28, 29). However, the application of the RSF algorithm to elucidate the high - dimensional, nonlinear dependencies within hematological profiles for UTUC prognostication remains unexplored.

To address the aforementioned issues, we conducted a large-scale, multicenter retrospective cohort study using the RSF algorithm to integrate preoperative systemic immune, inflammatory, and nutritional hematological indicators, establishing a novel prognostic signature (designated as the ML Score). The innovations of this study include: (1) the novel application of machine learning to the screening and modeling of hematological markers in UTUC; (2) the use of SHAP (SHapley Additive exPlanations) techniques to visualize the nonlinear threshold effects between key immune-inflammatory indicators and prognosis; and (3) a comprehensive evaluation of the score’s incremental value relative to traditional TNM staging across multiple independent external validation cohorts. Our objective is to develop a cost-effective, readily accessible, and biologically robust tool to assist clinicians in identifying occult high-risk patients and optimizing postoperative management strategies.

## 2 Methods

### 2.1 Study population and design

This retrospective, multicenter cohort study was conducted to develop and validate a novel prognostic model based on systemic immune-inflammatory hematological indicators. We collected data from four medical centers involving patients with upper tract urothelial carcinoma (UTUC) who underwent radical nephroureterectomy (RNU) between 2012 and 2024. The inclusion criteria were as follows: (1) postoperative histopathological confirmation of primary UTUC; (2) RNU performed as the primary treatment modality; (3) availability of complete preoperative hematological examination data; and (4) complete clinicopathological and follow-up data.

To minimize the interference of non-tumor factors on systemic immune-inflammatory markers and ensure the biological purity of the selected features, we established rigorous exclusion criteria. Patients were excluded if they had: (1) a history of other malignancies; (2) distant metastasis at the time of diagnosis; (3) received neoadjuvant chemotherapy or radiotherapy prior to surgery; (4) clinical evidence of acute infections, chronic inflammatory diseases, or autoimmune disorders; or (5) known hematological disorders or a history of blood transfusion within one month prior to surgery.

**Figure 1:**
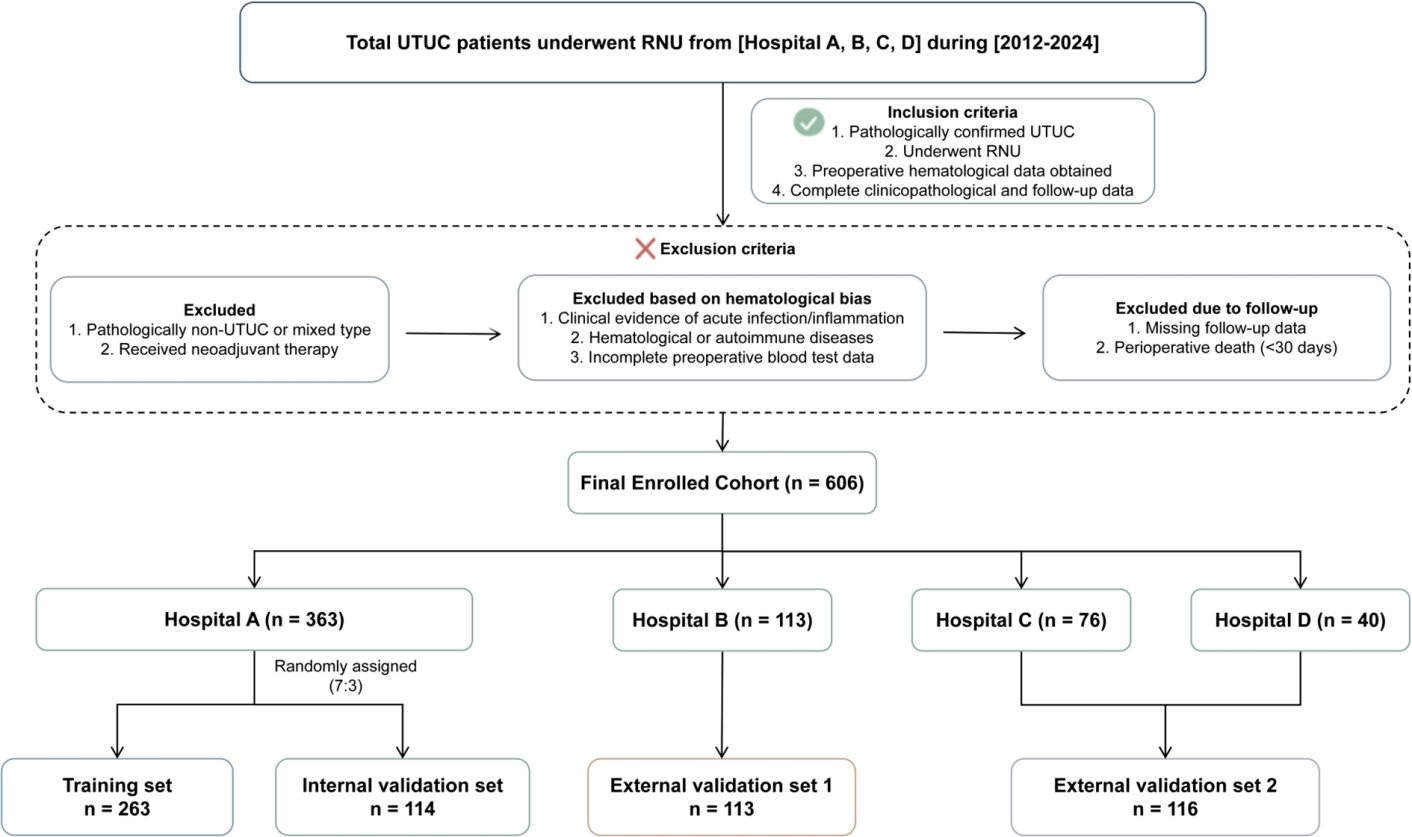
Flowchart of UTUC patient enrollment and study design. The diagram illustrates the inclusion and exclusion criteria applied to the initial population from four medical centers. A total of 606 patients with Upper Tract Urothelial Carcinoma (UTUC) were finally enrolled and divided into a training set (n = 263), an internal validation set (n = 114), and two external validation sets (n = 113 and n = 116).

The final cohort comprised 606 patients. To rigorously develop and validate the model, patients were divided into four cohorts: patients from The First Affiliated Hospital of Chongqing Medical University were randomly allocated in a 7:3 ratio to the training set (n = 263) and internal validation set (n = 114). Patients from the remaining three centers formed two independent external validation sets (External Validation Set 1: The Second Affiliated Hospital of Chongqing Medical University, n = 113; External Validation Set 2: Chongqing University Three Gorges Hospital and Chongqing University Fuling Hospital, n = 116 (**Figure 1**).

### 2.2 Data acquisition and endpoint specification

Demographic characteristics (age, gender) and lifestyle factors (history of smoking and alcohol consumption) were retrieved via a review of electronic medical records (EMRs). Detailed pathological features were collected, encompassing tumor size, laterality, location, pathological grade, pT stage (< pT2 vs. ≥ pT2), lymphovascular invasion (LVI), surgical margin status, and hydronephrosis. Tumor staging and grading were evaluated in accordance with the AJCC 8th edition TNM staging system and the WHO 2004/2016 classification system, respectively.

Regarding hematological parameters, 13 preoperative peripheral blood indicators associated with host inflammation and nutritional status were extracted, including neutrophil (NEUT), lymphocyte (LYM), monocyte (MONO), and platelet counts (PLT), as well as red blood cell distribution width (RDW). Additionally, composite inflammatory indices, such as the neutrophil - to - lymphocyte ratio (NLR), systemic immune - inflammation index (SII), and systemic inflammatory response index (SIRI), were computed. The primary endpoint of this study was disease-free survival (DFS), defined as the interval from the date of surgery to the first radiological or pathological confirmation of tumor recurrence (local or distant) or death from any cause.

### 2.3 Feature selection and machine learning modeling

**Figure 2:**
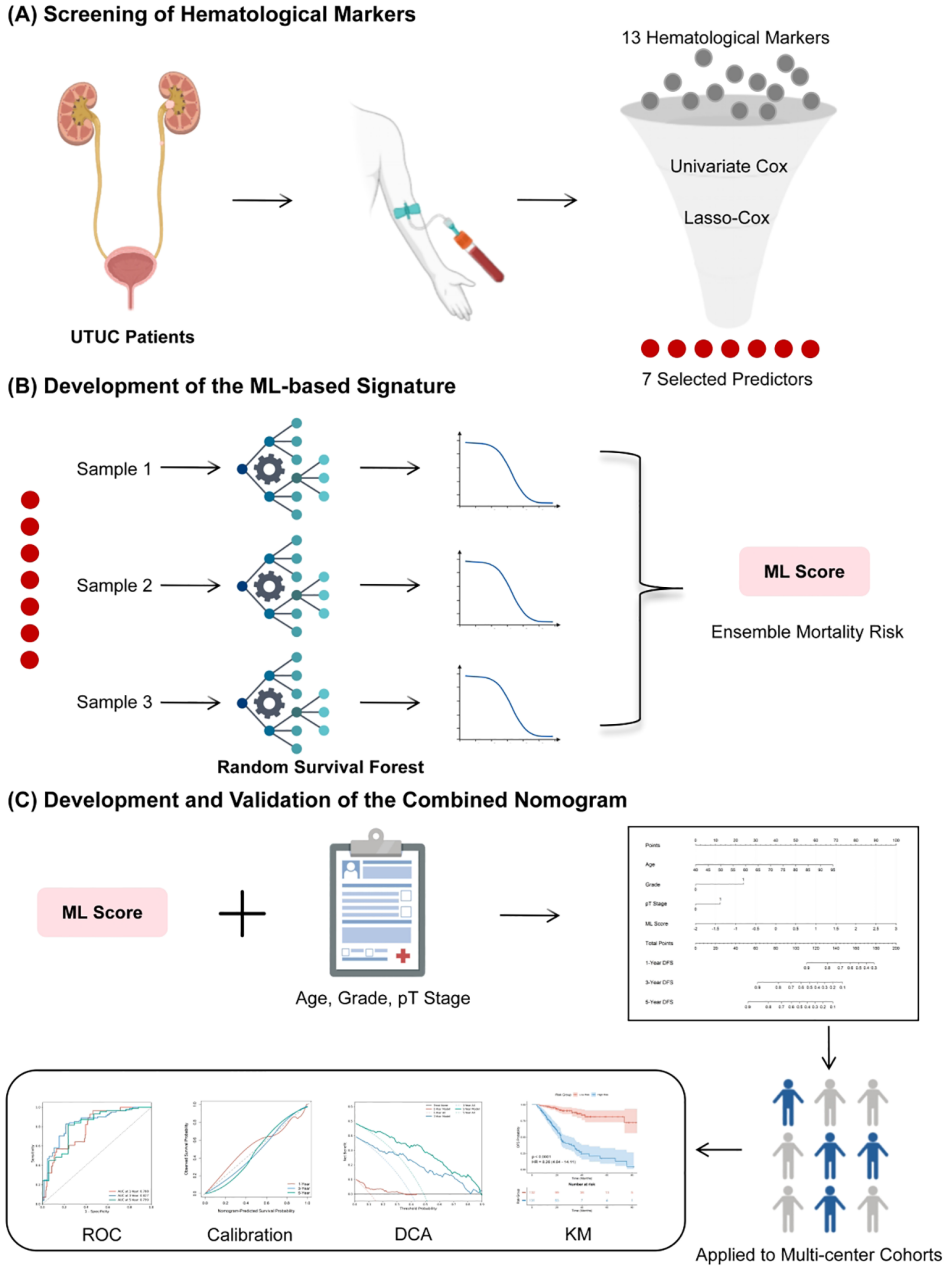
Schematic workflow of the machine learning-based hematological signature construction. **(A)** Data preprocessing and stepwise feature selection: 13 hematological markers were screened via univariate Cox and LASSO-Cox regression to identify 7 key predictors. **(B)** Development of the ML-based signature: A Random Survival Forest (RSF) algorithm was employed to construct the ML Score (Ensemble Mortality Risk). **(C)** Construction and validation of the combined nomogram: The ML Score was integrated with clinical risk factors (Age, Grade, pT Stage) to build a nomogram, which was comprehensively evaluated across multi-center cohorts.

To develop a machine learning (ML) signature that comprehensively reflects the host’s immune-inflammatory landscape, we implemented a “stepwise screening-ML modeling” strategy (**Figure 2**). To prevent data leakage, all feature screening and model training processes were strictly restricted to the training set. First, a univariate Cox regression analysis was performed to identify hematological indicators significantly associated with DFS (P < 0.05). Subsequently, the Least Absolute Shrinkage and Selection Operator (LASSO) Cox regression model was utilized to eliminate multicollinearity among variables and conduct dimensionality reduction. The optimal penalty parameter λ was ascertained through 10 - fold cross - validation.

Subsequently, the selected key features were fed into the Random Survival Forest (RSF) algorithm. RSF is an ensemble machine - learning approach specifically developed for right - censored survival data, which can effectively capture nonlinear associations and intricate interactions among variables (27). The RSF model was built using the scikit-survival library in Python (version 3.12). To optimize model performance while preventing overfitting, hyperparameter tuning was carried out strictly within the training set using a grid search strategy combined with 5 - fold cross - validation. The search space included the number of trees (n_estimators: 100, 200, 500), the minimum number of samples per leaf (min_samples_leaf: 5, 10, 20), the maximum tree depth (max_depth: 3, 5, None), and the maximum number of features (max_features: “sqrt”, “log2”). The optimal model was selected based on the highest cross - validated Concordance Index (C - index). The final hyperparameters were determined as follows: n_estimators = 200, min_samples_leaf = 20, max_depth = 5, and max_features = “sqrt”. This rigorous tuning process effectively restricted tree complexity and ensured robust generalizability across the validation cohorts. The cumulative hazard function (Ensemble Mortality) output by the RSF model was defined as the “ML Score” and standardized via Z-score transformation for clinical application. To enhance model interpretability, we employed the SHAP (SHapley Additive exPlanations) method to quantify each feature’s contribution to risk prediction and plotted SHAP dependence graphs to visualize the nonlinear threshold effects between key indicators (e.g., RDW, PLT) and prognosis risk.

### 2.4 Construction of the combined model (nomogram)

To evaluate the incremental prognostic value of the ML Score over traditional clinical indicators, we constructed a combined nomogram. In the training set, independent clinical risk factors were first identified through univariate and multivariate Cox regression analyses. Subsequently, the ML Score was incorporated with these independent clinical predictors (age, grade, and pT stage) into a multivariate Cox proportional hazards model. Based on this model, a prognostic nomogram was constructed using the rms package in R (version 4.4.2) to forecast the individual probabilities of 1-year, 3-year, and 5-year DFS.

### 2.5 Model performance assessment

The predictive performance of the models was comprehensively evaluated across the training cohort and three validation cohorts from multiple dimensions. Discrimination was measured using Harrell’s Concordance Index (C - index) and the area under the time - dependent receiver operating characteristic (ROC) curve (AUC). Calibration was visually evaluated by plotting calibration curves with bootstrap resampling to compare the predicted probabilities with the observed survival rates. Finally, the predictive accuracy of the combined nomogram was quantified using the C - index, and its clinical utility was assessed through Decision Curve Analysis (DCA) to determine the net benefit across a range of threshold probabilities.

### 2.6 Statistical analysis

All statistical analyses were carried out utilizing R software (version 4.4.2) and Python (version 3.12). A two - tailed P - value less than 0.05 was regarded as statistically significant. Continuous variables were compared via Student’s t - tests or Mann - Whitney U tests, whereas categorical variables were assessed using Chi - square tests or Fisher’s exact tests.

Subsequently, the continuous ML Scores generated in Python were imported into R for risk stratification and survival analysis. To translate the ML Score into a clinically actionable binary indicator, we utilized the surv_cutpoint function from the R package survminer. Based on the maximally selected rank statistics method, the optimal cutoff value was determined exclusively in the training set. This cutoff was then independently applied to all validation cohorts to stratify patients into high-risk and low-risk groups. Survival curves were constructed via the Kaplan - Meier approach, and the disparities between groups were analyzed by means of the Log - rank test. Subgroup analyses were carried out across the entire population to assess the robustness of the ML Score within different clinical strata, and these results were visualized through forest plots.

## 3 Results

### 3.1 Baseline characteristics

This retrospective study included 606 patients with upper tract urothelial carcinoma (UTUC) who underwent radical nephroureterectomy (RNU) at four medical centers. The detailed enrollment procedure and eligibility requirements are presented in **Figure 1**. The final cohort was partitioned into a training set (n = 263), an internal validation set (n = 114), and two independent external validation sets (External Validation Set 1, n = 113; External Validation Set 2, n = 116).

The baseline demographic and clinicopathological characteristics of patients across all cohorts are presented in detail in **Table 1**. The mean age of the entire cohort was 67.22 ± 9.59 years, and males accounted for 59.7% of the population. The training set and the three validation sets exhibited a balanced distribution in key demographic characteristics, including age (P = 0.381), gender (P = 0.612), smoking history (P = 0.209), and alcohol consumption history (P = 0.866), with no statistically significant differences. Moreover, tumor laterality (left vs. right, 56.8% vs. 43.2%) and tumor location (renal pelvis/ureter/both) were well - balanced across all cohorts.

**Table 1:** The baseline clinical characteristics of UTUC patients in four sets.

Characteristics	All (n = 606)	Training set (n =263)	Internal validation set (n = 114)	External validation set 1 (n = 113)	External validation set 2 (n =116)	*p*
Age	67.22 ± 9.59	66.53 ± 9.59	67.24 ± 11.01	67.76 ± 8.74	68.26 ± 8.85	0.381
Gender						0.612
Female	244 (40.3%)	100 (38.0%)	44 (38.6%)	50 (44.2%)	50 (43.1%)	
Male	362 (59.7%)	163 (62.0%)	70 (61.4%)	63 (55.8%)	66 (56.9%)	
Smoker						0.209
No	396 (65.3%)	167 (63.5%)	68 (59.6%)	79 (69.9%)	82 (70.7%)	
Yes	210 (34.7%)	96 (36.5%)	46 (40.4%)	34 (30.1%)	34 (29.3%)	
Drinker						0.866
No	442 (72.9%)	191 (72.6%)	81 (71.1%)	82 (72.6%)	88 (75.9%)	
Yes	164 (27.1%)	72 (27.4%)	33 (28.9%)	31 (27.4%)	28 (24.1%)	
Laterality						0.250
Left	344 (56.8%)	157 (59.7%)	61 (53.5%)	68 (60.2%)	58 (50.0%)	
Right	262 (43.2%)	106 (40.3%)	53 (46.5%)	45 (39.8%)	58 (50.0%)	
Tumor location						0.090
Renal Pelvis	304 (50.2%)	142 (54.0%)	63 (55.3%)	51 (45.1%)	48 (41.4%)	
Ureter	262 (43.2%)	110 (41.8%)	44 (38.6%)	51 (45.1%)	57 (49.1%)	
Renal Pelvis & Ureter	40 (6.6%)	11 (4.2%)	7 (6.1%)	11 (9.7%)	11 (9.5%)	
Size	2.90 (2.00-4.00)	3.00 (1.80-4.00)	3.00 (2.00-4.00)	2.00 (1.50-3.50)	3.00 (2.00-4.50)	0.003
pT Stage						0.170
< T2	217 (35.8%)	107 (40.7%)	35 (30.7%)	38 (33.6%)	37 (31.9%)	
≥ T2	389 (64.2%)	156 (59.3%)	79 (69.3%)	75 (66.4%)	79 (68.1%)	
Grade						0.001
Low	128 (21.1%)	39 (14.8%)	23 (20.2%)	36 (31.9%)	30 (25.9%)	
High	478 (78.9%)	224 (85.2%)	91 (79.8%)	77 (68.1%)	86 (74.1%)	
Lymphovascular invasion						0.153
No	552 (91.1%)	238 (90.5%)	106 (93.0%)	98 (86.7%)	110 (94.8%)	
Yes	54 (8.9%)	25 (9.5%)	8 (7.0%)	15 (13.3%)	6 (5.2%)	
Hydronephrosis						0.610
No	294 (48.5%)	123 (46.8%)	60 (52.6%)	58 (51.3%)	53 (45.7%)	
Yes	312 (51.5%)	140 (53.2%)	54 (47.4%)	55 (48.7%)	63 (54.3%)	
Margin						0.050
Negative	578 (95.4%)	256 (97.3%)	107 (93.9%)	103 (91.2%)	112 (96.6%)	
Positive	28 (4.6%)	7 (2.7%)	7 (6.1%)	10 (8.8%)	4 (3.4%)	

Nevertheless, notable statistical disparities were detected across cohorts regarding specific pathological indicators, which reflects the heterogeneity present in real - world data. Specifically, the median tumor diameter in External Validation Set 1 was the smallest (2.00 cm), being significantly smaller than that in the training set (3.00 cm) and other validation sets (P = 0.003). Moreover, the proportion of high - grade tumors in the training set was 85.2%, which was higher than that in External Validation Set 1 (68.1%) and External Validation Set 2 (74.1%) (P = 0.001). Additionally, disparities were also observed in the surgical margin positivity rates across cohorts (P = 0.05). This inter-cohort heterogeneity reflects real-world clinical variations and provides a rigorous scenario to test the model’s generalizability and robustness.

### 3.2 Selection of immune-inflammation-related hematological features

**Figure 3:**
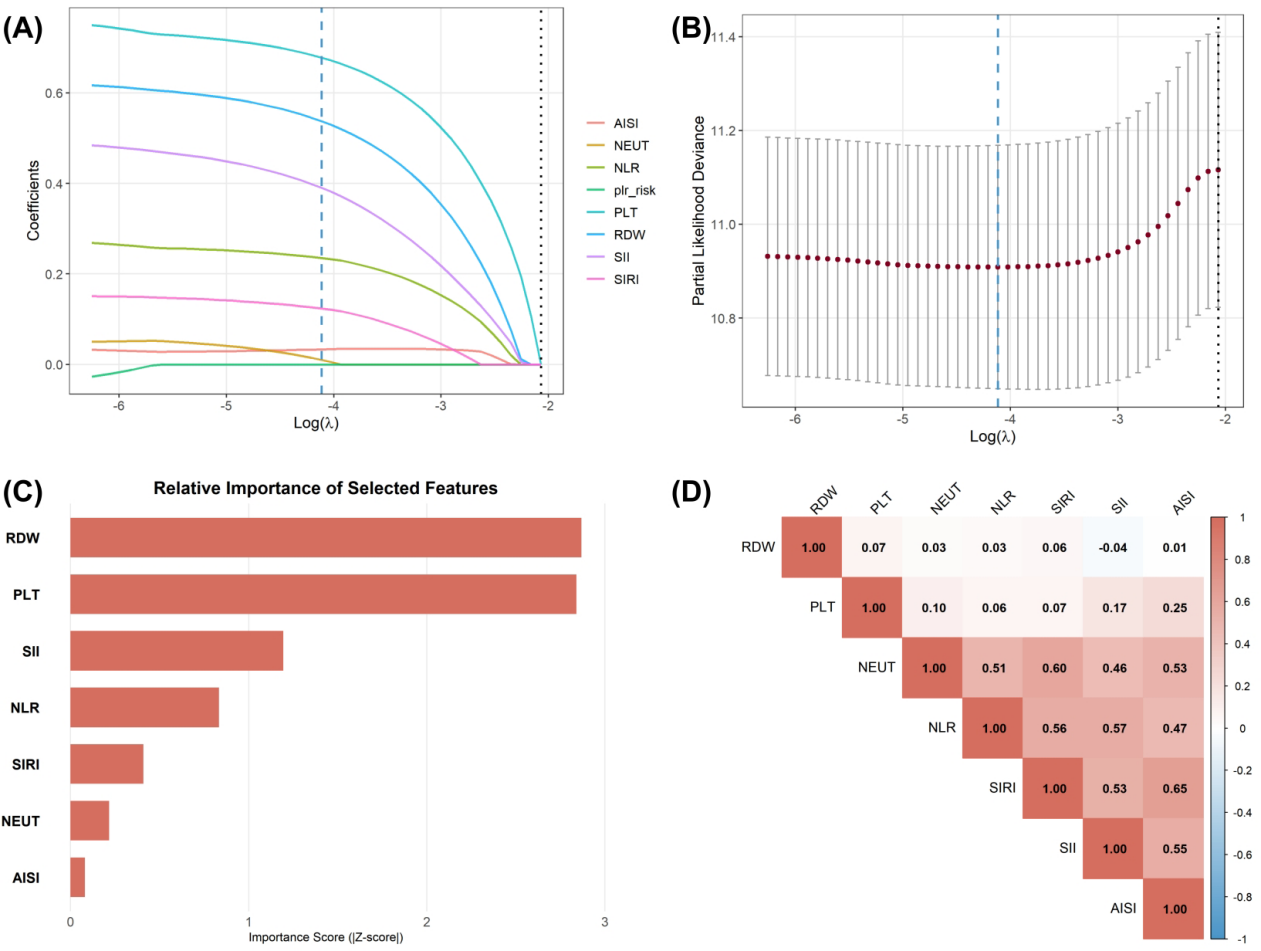
Screening of candidate hematological markers in the training cohort. **(A)** LASSO coefficient profiles of the 13 candidate hematological markers. **(B)** Selection of the optimal tuning parameter (λ) in the LASSO model using 10-fold cross-validation. The vertical dotted lines represent the optimal λ (min) and 1-SE λ. **(C)** Relative importance ranking of the 7 selected features based on the Z-score from the multivariate Cox regression model. RDW and PLT were identified as the top prognostic markers. **(D)** Pearson correlation heatmap of the 7 selected markers, indicating low collinearity among variables.

To establish prognostic markers reflecting the host’s systemic immune-inflammatory and nutritional status, we initially collected 13 preoperative hematological parameters. A univariate Cox regression analysis was performed to identify significantly associated indicators with DFS (P < 0.05). Subsequently, feature selection and dimensionality reduction were carried out using a LASSO - Cox regression model within the training dataset. The optimal penalty parameter λ was ascertained via 10 - fold cross - validation (**Figures 3A, B**). Ultimately, seven key predictors with non-zero coefficients were retained: RDW, PLT, NEUT, NLR, SIRI, SII, and AISI.

To further analyze the intrinsic relationships and contribution levels of these characteristics, we conducted correlation analysis and importance ranking. The Z-score ranking based on the multivariate Cox model (**Figure 3C**) showed that RDW (reflecting nutrition and inflammation) and PLT (reflecting coagulation and immune evasion) were identified as the two indicators with the highest prognostic weight for DFS. The Pearson correlation heatmap (**Figure 3D**) revealed that the absolute values of correlation coefficients among selected features were all below 0.7, indicating no severe multicollinearity among variables, making them suitable for simultaneous model inclusion. Additionally, the SHAP dependency graph based on the RSF model (**Supplementary Figure 1**) revealed a distinct nonlinear threshold effect between RDW, PLT, and mortality risk: when the standardized RDW value exceeded 0 or the PLT value surpassed 1, the SHAP values showed a steep upward trend. This discovery emphasizes the superiority of utilizing machine - learning algorithms compared to traditional linear models in capturing intricate nonlinear relationships within these data.

### 3.3 Construction and visualization of the ML score

**Figure 4:**
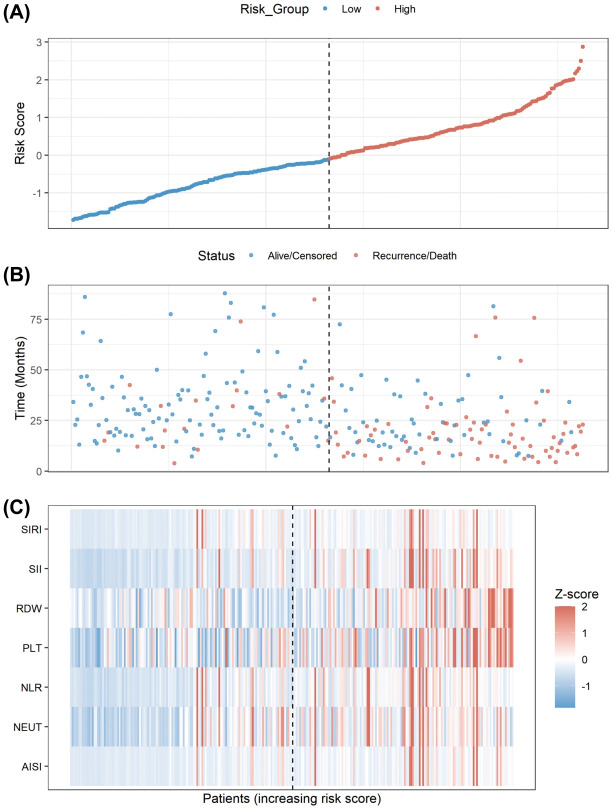
Visualization of the ML score distribution and its association with prognosis. **(A)** The distribution of the ML Score in the training set, classifying patients into low- (blue) and high-risk (red) groups based on the optimal cutoff. **(B)** The scatter plot showing survival status. Patients with higher risk scores exhibited a higher density of recurrence/death events (red dots). **(C)** A heatmap displaying the expression profiles of the 7 selected hematological markers. Increasing risk scores were associated with elevated levels of inflammation-related markers.

Based on the seven characteristics, we constructed an ML Score using the Random Survival Forest (RSF) algorithm and classified patients into low-risk and high-risk groups according to the optimal cutoff value determined by the training set. To visually demonstrate the ML score’s risk differentiation capability and underlying biological patterns, we plotted the risk factor association map in the training set (**Figure 4**).

As patients were ranked by risk score from low to high (**Figure 4A**), the scatter plot revealed a significant increase in recurrence/mortality event density within the high-risk group, with generally shorter recurrence durations (**Figure 4B**). The heatmap (**Figure 4C**) uncovered a distinct biological pattern: patients in the high-risk group generally show elevated expression levels of all included inflammation-related indicators, including RDW, NLR, and SII (colors shifted from blue to red). This result confirms at the individual level the strong association between a state of systemic hyperinflammation and immunosuppression and poor prognosis.

### 3.4 The prognostic value and independence verification of ML score

**Figure 5:**
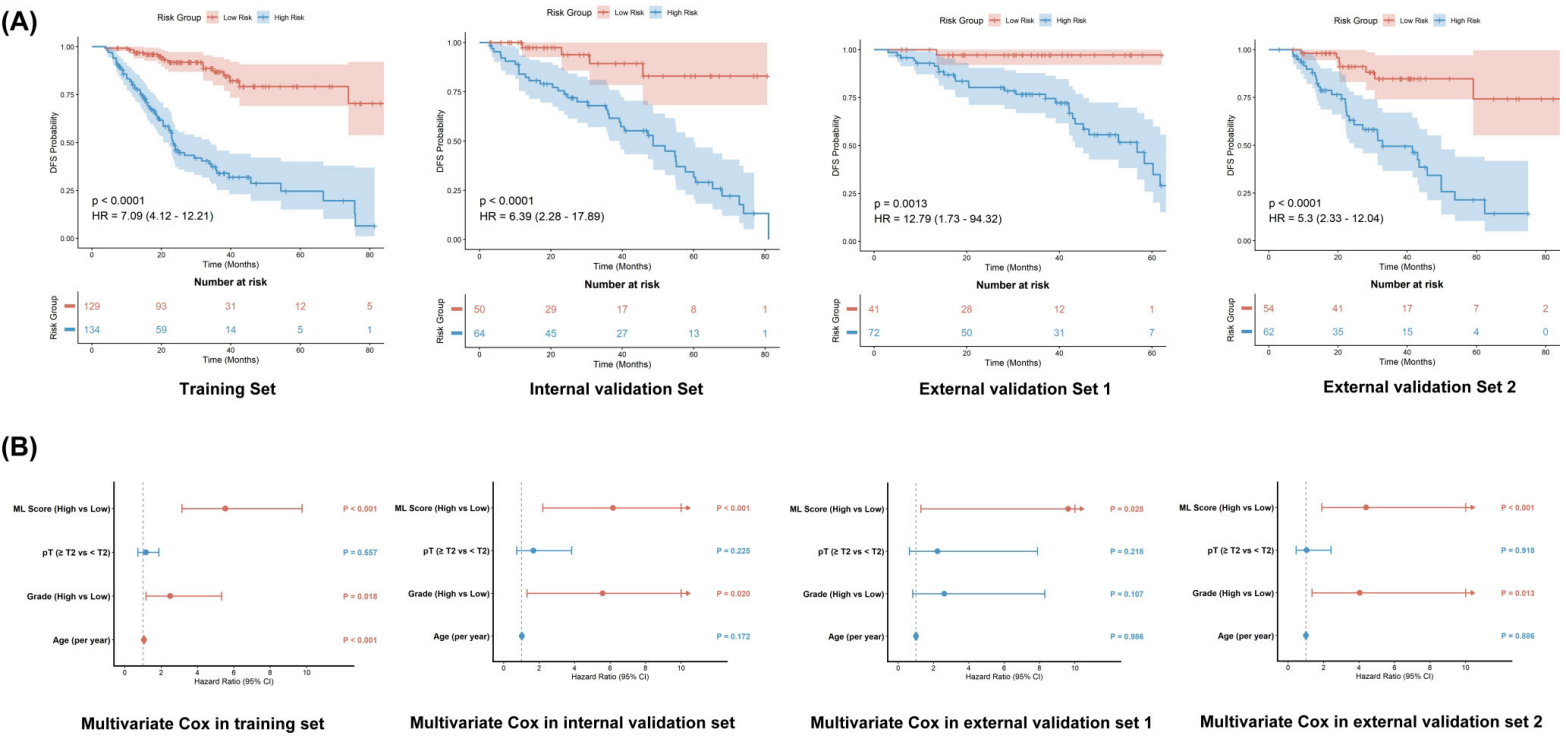
Prognostic value of the ML score in multiple cohorts. **(A)** Kaplan-Meier curves for disease-free survival (DFS) stratified by the ML Score (High vs. Low risk) across the training, internal validation, and two external validation sets. The ML Score demonstrated significant risk stratification capabilities (P < 0.05) in all cohorts. **(B)** Multivariate Cox regression analyses in each cohort, confirming that the ML Score (High vs. Low) remained an independent prognostic factor after adjusting for Age, Grade, and pT Stage.

Kaplan-Meier survival analysis (**Figure 5A**) demonstrated the ML Score’s robust risk stratification capability across all cohorts. In the training set, patients in the high-risk group showed significantly worse disease-free survival (DFS) than those in the low-risk group (HR = 7.09, 95% CI: 4.12-12.21, P < 0.0001). This significant discriminative power was further validated in the internal validation set (HR = 6.39, P <0.001) and two external validation sets (HR = 12.8 and 5.5, respectively; both P < 0.01), confirming the score’s excellent cross-center robustness.

To verify whether ML Score is independent of traditional clinical features, we first screened clinical pathological variables in the training set (**Table 2**). Multivariate Cox regression analysis identified Age, Grade, and pT stage as significant independent prognostic factors. Notably, lymphovascular invasion (LVI), often considered a risk factor, did not demonstrate independent statistical significance in our training cohort and was thus not included in the baseline clinical model. Subsequently, we included ML Score (High vs. Low) along with these three clinical factors in a multivariate Cox regression model (**Figure 5B**). The findings confirmed that, subsequent to adjustment for clinical confounding factors, the ML Score persisted as an independent prognostic risk factor for DFS. Significantly, within External Validation Set 1, the traditional pT stage and grade forfeited their statistical significance (P > 0.050), while the ML Score maintained significant independent prognostic value (P = 0.026). This implies that the ML Score might offer more reliable prognostic guidance compared to conventional pathological indicators in specific clinical contexts.

**Table 2:** Univariate and Multivariate Cox Regression Analysis of DFS.

Variables	Univariate Cox		Multivariate Cox	
	HR (95%CI)	*p*	HR (95%CI)	*p*
Gender				
Female	Reference			
Male	0.768 (0.501-1.175)	0.224		
Age, per 1-year increase	1.065 (1.040-1.090)	< 0.001	**1.069 (1.043-1.095)**	**< 0.001**
Smoker				
No	Reference			
Yes	1.019 (0.671-1.546)	0.931		
Drinker				
No	Reference			
Yes	1.176 (0.757-1.828)	0.471		
Size, per 1-cm increase	1.073 (0.962-1.198)	0.206		
Margin				
Negative	Reference			
Positive	1.621 (0.511-5.143)	0.412		
Lymphovascular invasion				
No	Reference		Reference	
Yes	2.050 (1.154-3.641)	0.014	1.505 (0.839-2.699)	0.170
Hydronephrosis				
No	Reference			
Yes	1.338 (0.883-2.029)	0.169		
Laterality				
Left	Reference			
Right	0.906 (0.593-1.384)	0.647		
Tumor location				
Renal Pelvis & Ureter	Reference			
Renal Pelvis	0.661 (0.263-1.658)	0.378		
Ureter	0.488 (0.189-1.260)	0.138		
Grade				
Low	Reference		Reference	
High	2.702 (1.305-5.595)	0.007	**2.471 (1.156-5.280)**	**0.020**
pT Stage				
< T2	Reference		Reference	
≥ T2	2.166 (1.379-3.402)	< 0.001	**1.672 (1.046-2.673)**	**0.032**

Bold values indicate statistical significance (*p* < 0.05).

**Figure 6:**
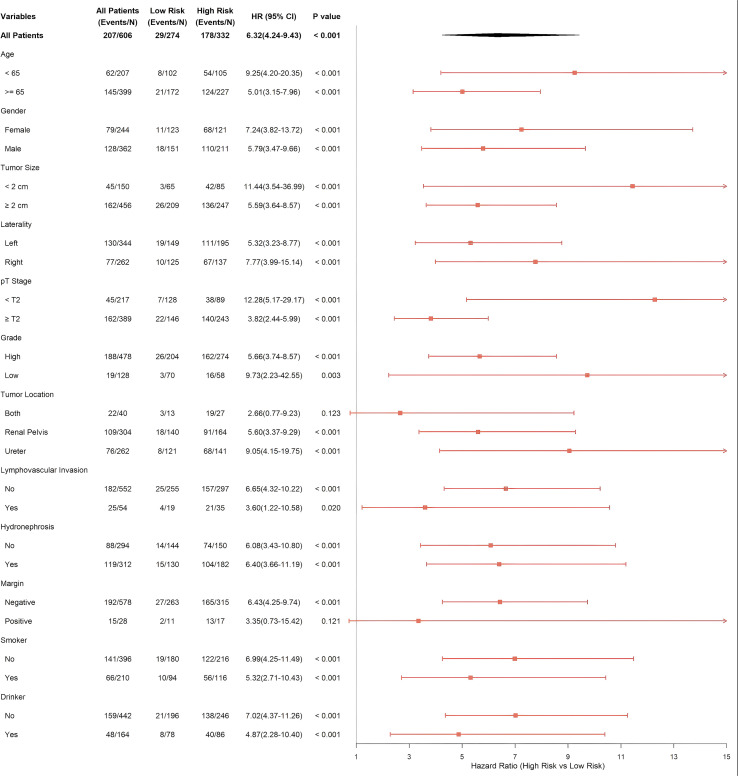
Subgroup analysis of the ML score in the total population. Forest plot displaying the Hazard Ratios (High vs. Low risk) of the ML Score in various clinical subgroups. The ML Score consistently showed significant prognostic value (HR > 1) across most subgroups, demonstrating its robustness. Arrows indicate that the confidence interval exceeds the axis limit.

Moreover, to comprehensively assess the applicability of the ML Score among diverse clinical populations, we performed in - depth subgroup analyses within the entire population (**Figure 6**). The results demonstrated that ML Score maintained consistent prognostic predictive power across most clinical subgroups demonstrating consistent performance without significant interaction. Specifically, high ML Score was significantly associated with poor prognosis in patients across all age groups (≤ 65 years vs. > 65 years), genders, tumor sizes, and locations (HR > 1 in all cases). Notably, ML Score exhibited exceptional discriminative power in subgroups traditionally considered “low-risk” in clinical practice. Even among patients with early-stage tumors (pT < T2), the high-risk group exhibited a 12.28-fold higher risk of recurrence compared to the low-risk group. In low-grade tumor patients, the HR reached 9.73.

Furthermore, to explicitly demonstrate the incremental methodological and clinical value of our machine learning framework, we systematically compared the predictive performance of the RSF - derived ML Score against both a conventional multivariable Cox proportional hazards model (constructed using the identical set of 7 hematological predictors) and widely - used single systemic inflammatory/nutritional indices (NLR, SII, SIRI, and AISI). As detailed in **Supplementary Table S1**, the ML Score consistently achieved an overall superior Concordance Index (C - index) across the training cohort and all three independent validation cohorts. Moreover, time - dependent AUC analysis revealed that while the predictive performance of simple unitary indices markedly deteriorated over long - term follow - up (e.g., 5 - year DFS AUCs exhibited a trend of decreased predictive stability), the ML Score generally maintained a more robust long - term predictive accuracy (5 - year AUC: 0.744).

### 3.5 Development and comparison of the combined model

**Figure 7:**
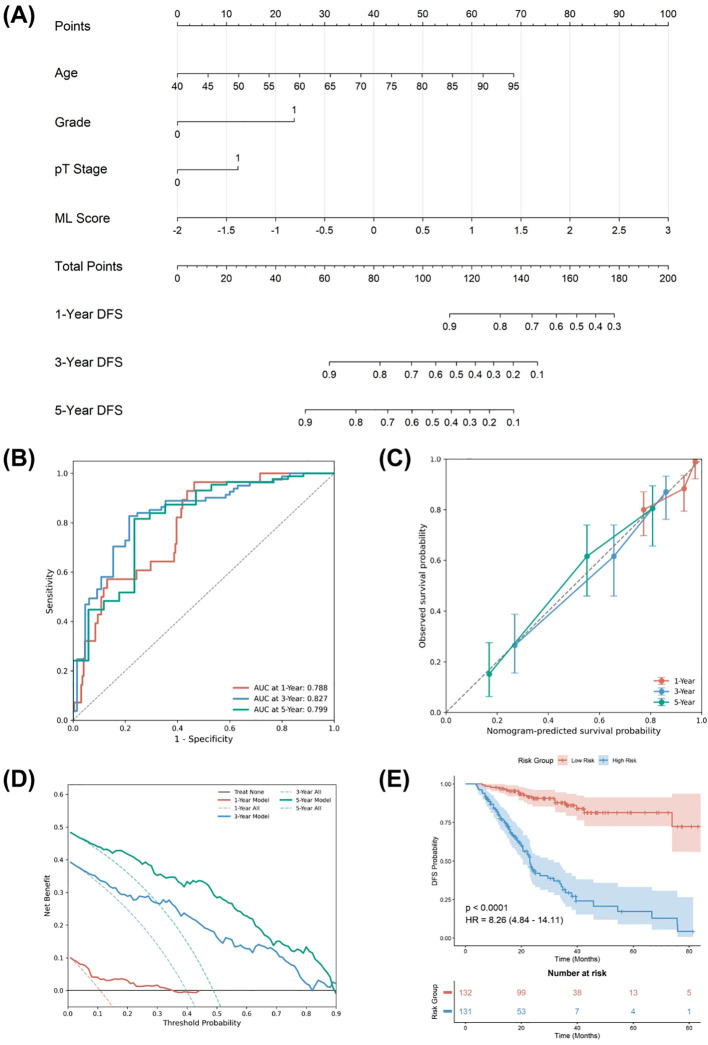
Development and performance of the combined nomogram in the training set. **(A)** The prognostic Nomogram (combined model) integrating the ML Score with Age, Grade, and pT Stage to predict 1-, 3-, and 5-year DFS probabilities. **(B)** Time-dependent ROC curves of the combined model. **(C)** Calibration curves comparing predicted and observed survival probabilities. **(D)** Decision Curve Analysis (DCA) illustrating the net benefit of the combined model. **(E)** Kaplan-Meier curve of the combined model (risk stratification).

Given the independent prognostic value of ML Score, we integrated it with independent clinical risk factors (Age, Grade, pT Stage) to construct a prognostic nomogram (**Figure 7A**) for quantifying the probability of 1-year, 3-year, and 5-year DFS. We comprehensively evaluated and compared the performance of the clinical model, the machine learning model, and the combined nomogram across all cohorts (**Table 3**). The model exhibited remarkable predictive accuracy, attaining a C - index of 0.762 in the training set. This high discriminatory capacity was consistently detected in the internal validation set (C - index = 0.752) and the two external validation sets (C - index = 0.761 and 0.712, respectively), corroborating the robust generalization of the immune - inflammatory signature among diverse clinical populations. Moreover, the time - dependent ROC curve indicated that the combined model sustained a high area under the curve (AUC) at all time points (**Figure 7B**). The calibration curve (**Figure 7C**) manifested an excellent concordance between the predicted probabilities and the actual observed probabilities. The decision curve analysis (DCA) decision curve (**Figure 7D**) further illustrated that the combined model conferred a superior net benefit for clinical decision - making compared to the clinical model across a broad spectrum of threshold probabilities. The Kaplan - Meier survival analysis based on the combined model (**Figure 7E**) also disclosed its exceptional proficiency in differentiating high - risk from low - risk patients in the training set (P < 0.001). Notably, the outstanding performance metrics, namely ROC, Calibration, and DCA, were consistently verified across the internal validation set (**Supplementary Figure 2**) and the two external validation sets (**Supplementary Figures 3**, **S4**), further substantiating the combined model’s robust generalization and stability.

**Table 3:** The performance of models in predicting DFS in UTUC patients.

Cohorts	Models	C-index	1-year AUC	3-year AUC	5-year AUC
Training Set	Clinical model	0.679 (0.615-0.743)	0.646 (0.536-0.762)	0.761 (0.681-0.836)	0.759 (0.628-0.864)
	ML model	0.741 (0.688-0.794)	0.795 (0.715-0.870)	0.777 (0.694-0.851)	0.744 (0.588-0.868)
	Combined model	0.762 (0.713-0.807)	0.788 (0.710-0.863)	0.827 (0.752-0.893)	0.799 (0.663-0.907)
Internal validation set	Clinical model	0.658 (0.548-0.764)	0.655 (0.450-0.823)	0.720 (0.575-0.858)	0.621 (0.458-0.786)
	ML model	0.729 (0.639-0.804)	0.746 (0.560-0.892)	0.725 (0.582-0.853)	0.755 (0.604-0.884)
	Combined model	0.752 (0.666-0.837)	0.759 (0.610-0.902)	0.784 (0.657-0.886)	0.764 (0.584-0.894)
External validation set 1	Clinical model	0.655 (0.543-0.755)	0.627 (0.395-0.821)	0.650 (0.460-0.805)	0.674 (0.412-0.922)
	ML model	0.718 (0.625-0.809)	0.776 (0.555-0.908)	0.699 (0.573-0.826)	0.662 (0.350-0.932)
	Combined model	0.761 (0.687-0.840)	0.786 (0.653-0.905)	0.733 (0.599-0.857)	0.740 (0.445-0.958)
External validation set 2	Clinical model	0.627 (0.532-0.729)	0.536 (0.335-0.733)	0.809 (0.695-0.904)	0.850 (0.702-0.962)
	ML model	0.705 (0.626-0.786)	0.711 (0.561-0.843)	0.738 (0.613-0.842)	0.771 (0.559-0.936)
	Combined model	0.712 (0.626-0.803)	0.674 (0.483-0.860)	0.838 (0.729-0.923)	0.856 (0.637-0.985)

## 4 Discussion

In this multicenter, large-scale retrospective study, we successfully developed and validated an ML Score—a machine learning-based signature integrating systemic immune-inflammatory and nutritional characteristics. While previous single-center studies have explored the prognostic value of hematological markers in UTUC using machine learning algorithms (30), to the best of our knowledge, this is the first multicenter validation study to address this topic using the Random Survival Forest (RSF) algorithm. This methodology effectively manages high - dimensional, nonlinear survival data. Meanwhile, the integration of SHAP values ensures model transparency and clinical interpretability. Our findings indicate that the ML Score functions as a robust and independent prognostic factor, effectively differentiating high - risk from low - risk patients across four independent cohorts with diverse clinical profiles. More importantly, the combined nomogram, which incorporates the ML Score with standard clinical parameters, exhibited satisfactory performance in terms of predictive accuracy, calibration, and clinical net benefit, serving as a reliable tool for individual risk assessment. These results underscore the necessity of incorporating host biological characteristics into existing staging systems to enhance risk stratification and personalized management for patients with UTUC.

The biological basis of the prognostic significance of the ML Score lies in its comprehensive depiction of the balance between “tumor-promoting inflammation” and “anti-tumor immunity” within the host (31, 32). Our model designates neutrophils and the neutrophil-to-lymphocyte ratio (NLR) as critical risk factors, which is consistent with prior research findings. Neutrophils not only secrete vascular endothelial growth factor (VEGF) and matrix metalloproteinases (MMPs) to promote tumor angiogenesis and invasion, but more importantly, recent studies have revealed that neutrophils can release Neutrophil Extracellular Traps (NETs). These reticular architectures entrap circulating tumor cells and protect them from immune elimination, thereby promoting distant metastasis (16, 18). Similarly, the significance of platelets (PLT) in our model validates the notion of a ‘platelet - tumor loop,’ in which platelets are hypothesized to encapsulate tumor cells, thereby establishing a physical and biochemical barrier that inhibits natural killer (NK) cell - mediated lysis (19). Notably, red blood cell distribution width (RDW) exhibits exceptional significance in our model. Beyond its indication of hematopoietic function, an elevated RDW is increasingly acknowledged as a marker of chronic inflammation and malnutrition (21, 24, 33). Consequently, the ML Score fundamentally assesses the systemic metabolic and immune burden exerted by tumors on the host, offering a distinctive biological perspective that supplements traditional anatomical staging.

Methodologically, a significant innovation of this study is the utilization of the Random Survival Forest (RSF) algorithm for feature integration, instead of the traditional Cox regression. The utility of advanced machine learning frameworks has been effectively demonstrated in deciphering complex urological and renal disease mechanisms, providing superior interpretability over traditional linear models (34). Biomedical data generally exhibits high complexity and frequently violates the linearity assumption of conventional statistical models. This limitation was strikingly evident in our comparative analysis (**Supplementary Table S1**). In this analysis, a conventional multivariable Cox model using the same 7 hematological predictors produced a relatively lower C - index of 0.605 in the training cohort, compared to the 0.741 achieved by the RSF model. By means of RSF modeling and SHAP (SHapley Additive exPlanations) analysis, we effectively identified the nonlinear relationships between hematological indicators and survival prognosis. For example, SHAP dependence plots clearly disclosed an “L-shaped” risk curve for RDW and an “S-shaped” risk curve for PLT. This indicates that these indicators may only trigger a sharp increase in mortality risk after exceeding specific biological thresholds—a subtle nonlinear effect that is easily underestimated or overlooked by traditional linear models (35, 36). Therefore, the apparent “machine learning gain” observed in our study is likely driven by the algorithm’s ability to accurately map these complex, non - linear host - tumor interactions. Additionally, the ensemble learning property of RSF (aggregating results from hundreds of decision trees) endows it with high robustness against noise and overfitting. This was confirmed in our subgroup analysis: even in subgroups traditionally considered “low-risk” (e.g., early-stage pT < T2 or low-grade tumors), the ML Score maintained significant discriminative power, demonstrating its ability to identify occult high-risk patients who might be undertreated.

Currently, preoperative risk assessment mainly relies on ureteroscopic biopsy and imaging to determine tumor grade and architecture, which guides the surgical approach (1). Postoperatively, it heavily relies on anatomical staging and pathological features (e.g., tumor stage, lymph node status, and lymphovascular invasion). While these conventional models are essential, they inherently focus on the “tumor” characteristics and largely neglect the “host” factor, specifically the systemic biological response to the malignancy. Our ML Score is not intended to replace these established anatomical staging systems, but rather to serve as a crucial biological supplement. Its main advantage lies in its non - invasive nature and accessibility, as it is entirely derived from routine, low - cost preoperative peripheral blood tests. By integrating these hematological markers, the ML Score introduces the previously absent dimension of “host systemic immune - nutritional reserve” into the risk evaluation framework. Clinically, its intended application is to identify occult high - risk patients who might be clinically under - staged by traditional anatomical criteria alone. For example, in patients with intermediate - risk pathology or equivocal clinical presentations, a high ML Score could tip the balance towards recommending more aggressive adjuvant therapies or implementing intensified postoperative surveillance protocols, thereby refining personalized management in UTUC.

The combined model developed in this study possesses substantial clinical translational significance. Statistical analyses indicated that the combined model attained a high C - index across multiple cohorts (ranging from 0.712 to 0.762), and an improved clinical net benefit was observed in Decision Curve Analysis (DCA). Considering that this score is entirely derived from routine preoperative blood examinations, its low cost and high accessibility render it highly feasible for clinical application. In clinical practice, current indications for adjuvant chemotherapy in patients with intermediate-risk disease (e.g., pT2/pT3 stage) remain controversial (1, 37). Consequently, our ML Score serves as a biologically grounded stratification tool. For patients presenting with clinically equivocal intermediate - risk diseases, a high ML Score indicates an unfavorable immune - nutritional milieu, which may potentially warrant more aggressive adjuvant therapeutic approaches or participation in immunotherapy clinical trials focused on the inflammatory microenvironment. Moreover, patients classified as high - risk through the nomogram ought to receive more intensive postoperative monitoring, such as an elevated frequency of cystoscopy or imaging, to expedite the early detection of recurrence. Furthermore, evaluating the host’s systemic immune and nutritional landscape is particularly crucial, considering the typical demographic profile of UTUC patients, who are often elderly and have multiple concurrent morbidities. Age - related comorbidities and underlying frailty can significantly impair a patient’s ability to tolerate radical surgery or traditional platinum - based adjuvant chemotherapy. Recently, the therapeutic paradigm for urothelial carcinoma has rapidly evolved with the emergence of novel therapies, including immune checkpoint inhibitors and targeted agents, which provide critical alternatives for elderly or frail patients (38, 39). In this context, our ML Score could serve as a valuable surrogate marker for the host’s physiological reserve and immune - inflammatory baseline. By accurately stratifying patients, the ML Score may assist clinicians in weighing oncological risks against the competing risks of age - related comorbidities, thus optimizing patient selection for these novel therapies and refining personalized management strategies in the aging UTUC population.

Although the findings are promising, it is necessary to recognize several limitations. Firstly, as a retrospective study, despite the implementation of strict inclusion and exclusion criteria and a multicenter design to minimize bias, selection bias is still inevitable. Moreover, the exclusion of patients due to missing follow - up data may undermine the representativeness of the results. Secondly, this study only analyzed hematological indicators at a single preoperative time point. Nevertheless, the immune - inflammatory status is dynamic, and a single measurement may be affected by transient physiological states (e.g., dehydration or stress), thus failing to capture longitudinal variations. Future studies integrating postoperative dynamic monitoring could further enhance predictive accuracy. Thirdly, despite efforts to standardize data, objective heterogeneity in laboratory equipment, calibration standards, and surgical techniques (e.g., scope of lymph node dissection) among medical centers may introduce potential batch effects. Although the model exhibited robustness in external validation, prudence is required when generalizing the results to a wider population. Fourth, while we proposed potential mechanisms of immune evasion and inflammatory responses based on the literature, this study did not conduct molecular biology experiments on tissue samples to directly validate these pathways. Fifthly, the methodological approach employed to dichotomize the continuous ML Score warrants consideration. We used the maximally selected rank statistics (surv_cutpoint function) to determine the optimal cutoff. While this data - driven approach is practical, it is inherently sensitive to the specific distribution of the training cohort and bears the theoretical risk of overestimating prognostic performance. Although we strictly applied this predefined cutoff to three independent validation cohorts and observed consistent risk stratification, the potential for cohort - specific bias cannot be entirely ruled out. In future prospective validations, alternative strategies should be explored to establish a more universally applicable threshold. For instance, the X - tile method could be employed to identify optimal risk cut - offs across diverse populations, or if a robust optimal cut - off cannot be established universally, a median split approach could simply be adopted. Finally, this study lacks data on emerging biomarkers such as molecular subtypes (e.g., FGFR3 mutations) or PD-L1 expression (40–42). Future efforts to integrate genomic features with our hematological data may lead to the development of more sophisticated multimodal predictive models.

## 5 Conclusion

In conclusion, this study developed and validated a reliable machine learning (ML)-based global immune-inflammatory and nutritional signature score. By considering the complex nonlinear interactions between the host’s biological state and tumor progression, the ML score demonstrates significant incremental prognostic value in comparison to traditional anatomical staging. The proposed combined model provides UTUC patients a straightforward and precise individualized risk stratification tool, which is anticipated to aid in clinical decision - making and optimize postoperative management strategies.

## Data Availability

The original contributions presented in the study are included in the article/**Supplementary Material**. Further inquiries can be directed to the corresponding authors.

## References

[1] Masson-Lecomte A Birtle A Pradere B Capoun O Compérat E Domínguez-Escrig J . European Association of Urology Guidelines on upper urinary tract urothelial carcinoma: Summary of the 2025 update. Eur Urol. (2025) 87:697–716. doi: 10.1016/j.eururo.2025.02.023. PMID: 40118741

[2] Cho J Ha S Kim S Sung H Kwon G . Prognostic significance of epithelial-mesenchymal transition phenotypes in upper urinary tract urothelial carcinoma. Pathol Res Pract. (2018) 214:547–54. doi: 10.1016/j.prp.2018.02.007. PMID: 29572121

[3] Basile G Bandini M Li R Poch M Necchi A Spiess P . Gold standard nephroureterectomy, chemoprophylaxis and surveillance in upper tract urothelial carcinoma. Curr Opin Urol. (2025) 35:75–82. doi: 10.1097/mou.0000000000001247. PMID: 39529478

[4] Zhou L Huang C Sun S Ning K Tang S . Kidney sparing surgery versus radical nephroureterectomy in upper tract urothelial carcinoma: A meta-analysis and systematic review. Front Oncol. (2025) 15:1448079. doi: 10.3389/fonc.2025.1448079. PMID: 40242251 PMC11999840

[5] Mertens L Sharma V Matin S Boorjian S Houston Thompson R van Rhijn B . Bladder recurrence following upper tract surgery for urothelial carcinoma: A contemporary review of risk factors and management strategies. Eur Urol Open Sci. (2023) 49:60–6. doi: 10.1016/j.euros.2023.01.004. PMID: 36793750 PMC9922921

[6] Wang L Chou W Pang S Yang C Chuang C Chang Y . Risk stratification of upper urinary tract urothelial carcinoma patients for survival prediction: A simple summation scoring method. J Cancer. (2018) 9:2284–94. doi: 10.7150/jca.24815. PMID: 30026823 PMC6036706

[7] Li C Yang J Xu F Han D Zheng S Kaaya R . A prognostic nomogram for the cancer-specific survival of patients with upper-tract urothelial carcinoma based on the surveillance, epidemiology, and end results database. BMC Cancer. (2020) 20:534. doi: 10.1186/s12885-020-07019-5. PMID: 32513124 PMC7282122

[8] Roscigno M Cha E Rink M Seitz C Novara G Chromecki T . International validation of the prognostic value of subclassification for Ajcc stage Pt3 upper tract urothelial carcinoma of the renal pelvis. BJU Int. (2012) 110:674–81. doi: 10.1111/j.1464-410X.2012.10930.x. PMID: 22348322

[9] Guan B Tang S Zhan Y Li Y Fang D Peng D . Prognostic performance of the 1973 and 2004 Who grading classification in upper tract urothelial carcinoma. Urol Oncol. (2019) 37:529.e19–.e25. doi: 10.1016/j.urolonc.2019.01.013. PMID: 31153747

[10] Huynh T Wei X Arulshankar S Huang J Rajarubendra N Chu K . Accuracy and limitations of ureteroscopic biopsy in the staging and grading of upper tract urothelial carcinoma: A retrospective analysis at a large tertiary center. Bladder (San Franc). (2025) 12:e21200048. doi: 10.14440/bladder.2025.0006. PMID: 40933475 PMC12417869

[11] Hanahan D . Hallmarks of cancer: New dimensions. Cancer Discov. (2022) 12:31–46. doi: 10.1158/2159-8290.Cd-21-1059. PMID: 35022204

[12] Wang Y Hao X Li G . Prognostic and clinical pathological significance of the systemic immune-inflammation index in urothelial carcinoma: A systematic review and meta-analysis. Front Oncol. (2024) 14:1322897. doi: 10.3389/fonc.2024.1322897. PMID: 38595827 PMC11002112

[13] Diakos C Charles K McMillan D Clarke S . Cancer-related inflammation and treatment effectiveness. Lancet Oncol. (2014) 15:e493–503. doi: 10.1016/s1470-2045(14)70263-3. PMID: 25281468

[14] Kiss M Halasz L Hadadi E Berger W Tzerpos P Poliska S . Epigenomic preconditioning of peripheral monocytes determines their transcriptional response to the tumor microenvironment. Genome Med. (2025) 17:82. doi: 10.1186/s13073-025-01511-y. PMID: 40702585 PMC12285133

[15] Yu J Zhang Y Ho M Kam K Rong S Young A . Integrated roles of systemic diseases and serum biomarkers in the incidence of age-related macular degeneration: The UK Biobank study. Med Bull. (2025) 1:188–99. doi: 10.1002/mdb2.70008. PMID: 41925078

[16] Hedrick C Malanchi I . Neutrophils in cancer: Heterogeneous and multifaceted. Nat Rev Immunol. (2022) 22:173–87. doi: 10.1038/s41577-021-00571-6. PMID: 34230649

[17] Ozel I Duerig I Domnich M Lang S Pylaeva E Jablonska J . The good, the bad, and the ugly: Neutrophils, angiogenesis, and cancer. Cancers (Basel). (2022) 14(3):536. doi: 10.3390/cancers14030536. PMID: 35158807 PMC8833332

[18] Cools-Lartigue J Spicer J McDonald B Gowing S Chow S Giannias B . Neutrophil extracellular traps sequester circulating tumor cells and promote metastasis. J Clin Invest. (2013) 123:3446–58. doi: 10.1172/jci67484. PMID: 23863628 PMC3726160

[19] Placke T Örgel M Schaller M Jung G Rammensee H Kopp H . Platelet-derived Mhc class I confers a pseudonormal phenotype to cancer cells that subverts the antitumor reactivity of natural killer immune cells. Cancer Res. (2012) 72:440–8. doi: 10.1158/0008-5472.Can-11-1872. PMID: 22127925

[20] Jiang P Gu S Pan D Fu J Sahu A Hu X . Signatures of T cell dysfunction and exclusion predict cancer immunotherapy response. Nat Med. (2018) 24:1550–8. doi: 10.1038/s41591-018-0136-1. PMID: 30127393 PMC6487502

[21] Weiss G Ganz T Goodnough L . Anemia of inflammation. Blood. (2019) 133:40–50. doi: 10.1182/blood-2018-06-856500. PMID: 30401705 PMC6536698

[22] Salvagno G Sanchis-Gomar F Picanza A Lippi G . Red blood cell distribution width: A simple parameter with multiple clinical applications. Crit Rev Clin Lab Sci. (2015) 52:86–105. doi: 10.3109/10408363.2014.992064. PMID: 25535770

[23] Förhécz Z Gombos T Borgulya G Pozsonyi Z Prohászka Z Jánoskuti L . Red cell distribution width in heart failure: Prediction of clinical events and relationship with markers of ineffective erythropoiesis, inflammation, renal function, and nutritional state. Am Heart J. (2009) 158:659–66. doi: 10.1016/j.ahj.2009.07.024. PMID: 19781428

[24] Haenggi E Kaegi-Braun N Wunderle C Tribolet P Mueller B Stanga Z . Red blood cell distribution width (Rdw) - a new nutritional biomarker to assess nutritional risk and response to nutritional therapy? Clin Nutr. (2024) 43:575–85. doi: 10.1016/j.clnu.2024.01.001. PMID: 38242035

[25] Zheng Y Yu D Yu Z Zhao D Chen Y Chen W . Association of preoperative systemic immune-inflammation index and prognostic nutritional index with survival in patients with upper tract urothelial carcinoma. J Cancer. (2020) 11:5665–77. doi: 10.7150/jca.44915. PMID: 32913461 PMC7477451

[26] Setti Boubaker N Saidani B Saadi A Mokadem S Naimi Z Kochbati L . Deciphering the efficiency of preoperative systemic-immune inflammation related markers in predicting oncological outcomes of upper tract urothelial carcinoma patients after radical nephroureterectomy. Investig Clin Urol. (2025) 66:194–206. doi: 10.4111/icu.20250044. PMID: 40312899 PMC12058541

[27] Beam A Kohane I . Big data and machine learning in health care. Jama. (2018) 319:1317–8. doi: 10.1001/jama.2017.18391. PMID: 29532063

[28] Ishwaran H Kogalur U Blackstone E Lauer M . Random survival forests. Ann Appl Stat. (2008) 2:841–60, 20. doi: 10.1201/9781003493679-19

[29] Kourou K Exarchos T Exarchos K Karamouzis M Fotiadis D . Machine learning applications in cancer prognosis and prediction. Comput Struct Biotechnol J. (2015) 13:8–17. doi: 10.1016/j.csbj.2014.11.005. PMID: 25750696 PMC4348437

[30] Liu J Wu P Lai S Wang J Hou H Zhang Y . Prognostic models for upper urinary tract urothelial carcinoma patients after radical nephroureterectomy based on a novel systemic immune-inflammation score with machine learning. BMC Cancer. (2023) 23:574. doi: 10.1186/s12885-023-11058-z. PMID: 37349696 PMC10286456

[31] Mantovani A Allavena P Sica A Balkwill F . Cancer-related inflammation. Nature. (2008) 454:436–44. doi: 10.1038/nature07205. PMID: 18650914

[32] Greten F Grivennikov S . Inflammation and cancer: Triggers, mechanisms, and consequences. Immunity. (2019) 51:27–41. doi: 10.1016/j.immuni.2019.06.025. PMID: 31315034 PMC6831096

[33] Cao W Shao Y Wang N Jiang Z Yu S Wang J . Pretreatment red blood cell distribution width may be a potential biomarker of prognosis in urologic cancer: A systematic review and meta-analysis. biomark Med. (2022) 16:1289–300. doi: 10.2217/bmm-2022-0409. PMID: 36912229

[34] Wu Y Liang Y Liang J Sun W Zhang Q Li M . Systematic deciphering of ATBC nephrotoxicity mechanisms via machine learning and single-cell analysis. Med Bull. (2026) 2:141–54. doi: 10.1002/mdb2.70027. PMID: 41925078

[35] Lundberg S Erion G Chen H DeGrave A Prutkin J Nair B . From local explanations to global understanding with explainable Ai for trees. Nat Mach Intell. (2020) 2:56–67. doi: 10.1038/s42256-019-0138-9. PMID: 32607472 PMC7326367

[36] Deo R . Machine learning in medicine. Circulation. (2015) 132:1920–30. doi: 10.1161/circulationaha.115.001593. PMID: 26572668 PMC5831252

[37] Birtle A Johnson M Chester J Jones R Dolling D Bryan R . Adjuvant chemotherapy in upper tract urothelial carcinoma (the Pout trial): A phase 3, open-label, randomised controlled trial. Lancet. (2020) 395:1268–77. doi: 10.1016/s0140-6736(20)30415-3. PMID: 32145825 PMC7181180

[38] Ferro M Chiujdea S Vartolomei M Bove P Porreca A Busetto G . Advanced age impacts survival after radical nephroureterectomy for upper tract urothelial carcinoma. Clin Genitourin Cancer. (2024) 22:27–37. doi: 10.1016/j.clgc.2023.08.001. PMID: 37661507

[39] Ferro M Crocetto F Tataru S Barone B Dolce P Lucarelli G . Predictors of efficacy of immune checkpoint inhibitors in patients with advanced urothelial carcinoma: A systematic review and meta-analysis. Clin Genitourin Cancer. (2023) 21:574–83. doi: 10.1016/j.clgc.2023.05.017. PMID: 37419854

[40] Fujii Y Sato Y Suzuki H Kakiuchi N Yoshizato T Lenis A . Molecular classification and diagnostics of upper urinary tract urothelial carcinoma. Cancer Cell. (2021) 39:793–809.e8. doi: 10.1016/j.ccell.2021.05.008. PMID: 34129823 PMC9110171

[41] Kim K Alam S Kuo F Chen Z Yip W Katims A . Molecular heterogeneity and immune infiltration drive clinical outcomes in upper tract urothelial carcinoma. Eur Urol. (2025) 87:342–54. doi: 10.1016/j.eururo.2024.10.024. PMID: 39550333 PMC12092068

[42] Lu Y Kang J Luo Z Song Y Tian J Li Z . The prevalence and prognostic role of Pd-L1 in upper tract urothelial carcinoma patients underwent radical nephroureterectomy: A systematic review and meta-analysis. Front Oncol. (2020) 10:1400. doi: 10.3389/fonc.2020.01400. PMID: 32974145 PMC7472102

