# Supplementary material for: Machine learning-based integration of systemic immune-inflammation and nutritional signatures for predicting disease-free survival in upper tract urothelial carcinoma: a multicenter study
Source: Front Immunol. 2026 Apr 22;17:1759547. doi: 10.3389/fimmu.2026.1759547 (PMC13143977; doi:10.3389/fimmu.2026.1759547)
Supplement: Supplementary file 1 [file DataSheet1.docx]

Supplementary Material

# Supplementary Figures and Tables

## Supplementary Figures

##
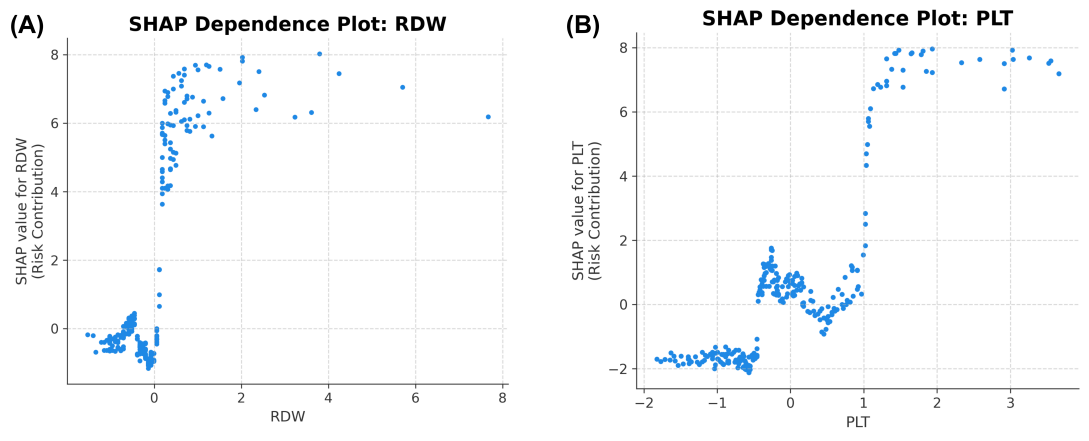


## Supplementary Figure 1. Non-linear relationship between key hematological markers and prognosis. SHAP dependence plots for (A) RDW and (B) PLT derived from the Random Survival Forest model. The x-axis represents the standardized feature value, and the y-axis represents the SHAP value (impact on risk). Both markers exhibited distinct non-linear threshold effects on survival risk.

##
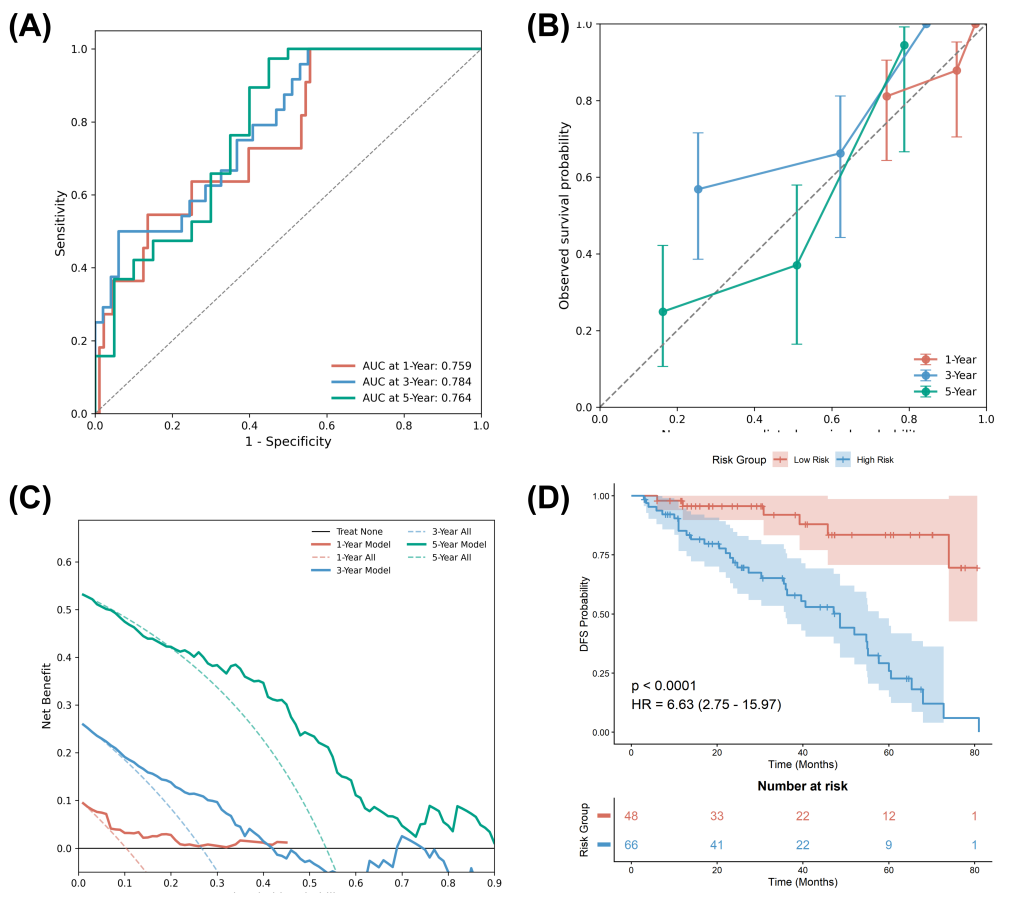


**Supplementary Figure 2.** **Performance evaluation of the combined model in the Internal Validation Set.** (A) Time-dependent ROC curves. (B) Calibration curves. (C) Decision Curve Analysis (DCA). (D) Kaplan-Meier survival analysis based on the combined model.


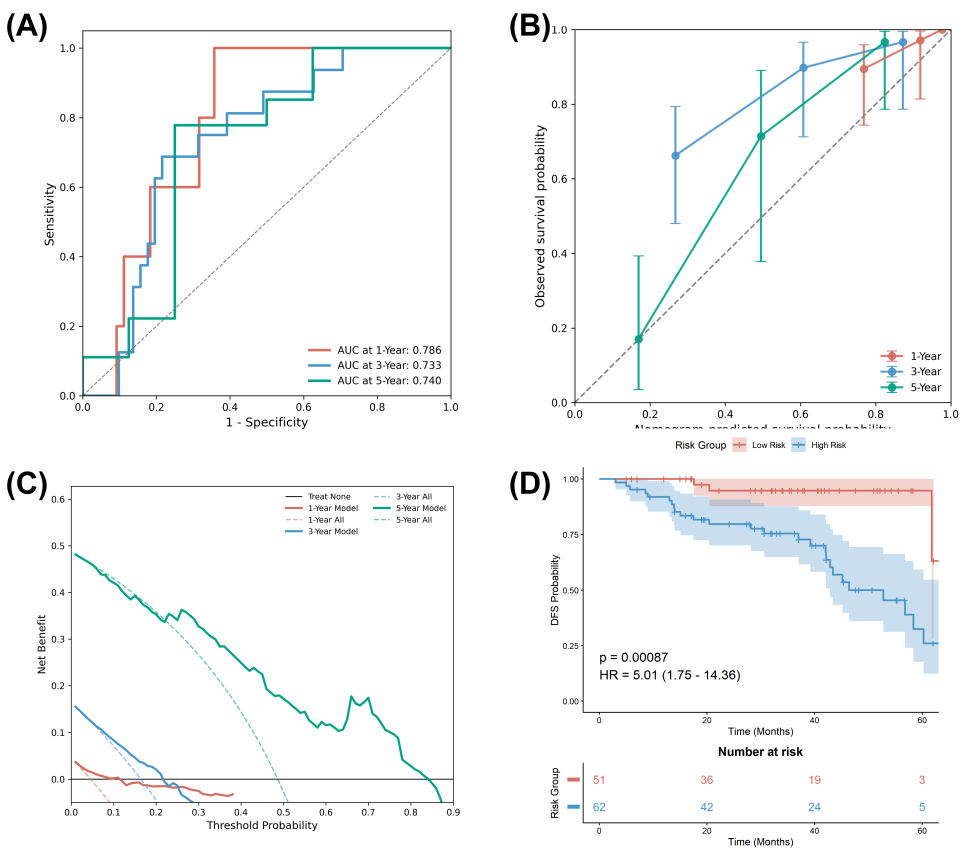


**Supplementary Figure 3.** **Performance evaluation of the combined model in the External Validation Set 1.** (A) Time-dependent ROC curves. (B) Calibration curves. (C) Decision Curve Analysis (DCA). (D) Kaplan-Meier survival analysis based on the combined model.


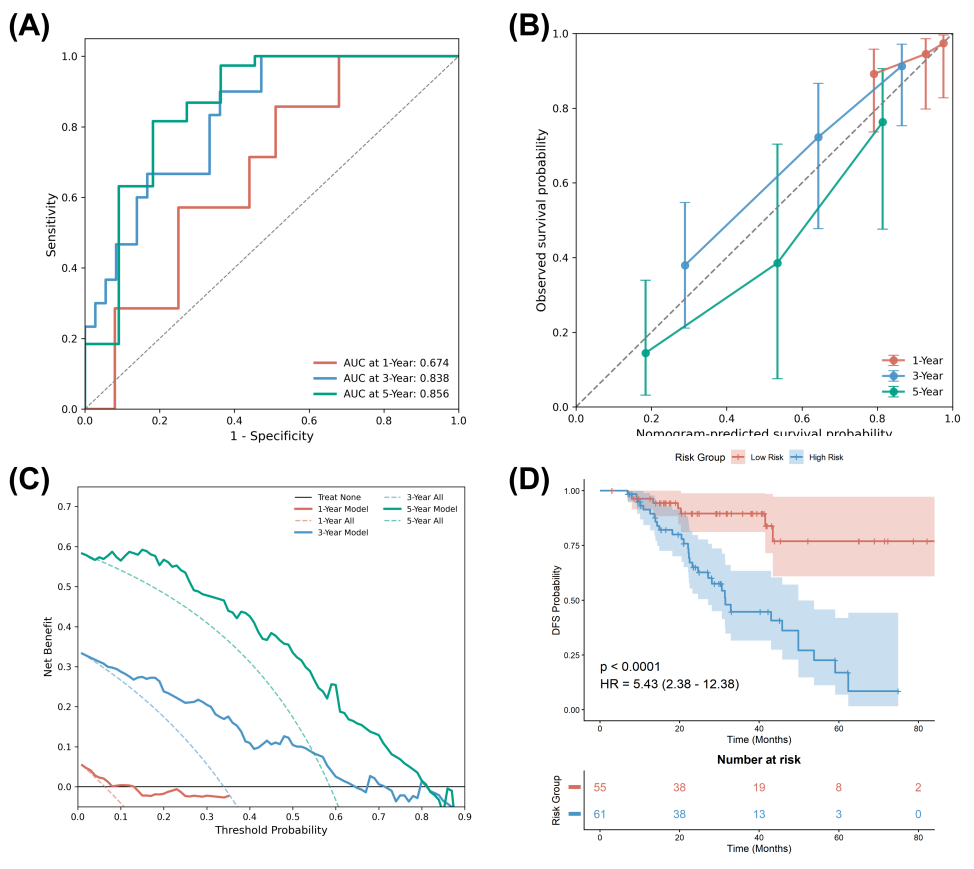


**Supplementary Figure 4.** **Performance evaluation of the combined model in the External Validation Set 2.** (A) Time-dependent ROC curves. (B) Calibration curves. (C) Decision Curve Analysis (DCA). (D) Kaplan-Meier survival analysis based on the combined model.

## Supplementary Table

**Table S1. Comprehensive performance comparison of the ML Score against a conventional multivariable Cox model and single inflammatory/nutritional indices.**

| **Cohorts** | **Models** | **C-index** | **1-year AUC** | **3-year AUC** | **5-year AUC** |
| --- | --- | --- | --- | --- | --- |
| Training Set | ML Score | 0.741 (0.688-0.794) | 0.795 (0.715-0.870) | 0.777 (0.694-0.851) | 0.744 (0.588-0.868) |
|  | Conventional Cox | 0.605 (0.542-0.670) | 0.674 (0.569-0.777) | 0.641 (0.544-0.733) | 0.567 (0.415-0.739) |
|  | NLR | 0.549 (0.489-0.613) | 0.568 (0.457-0.665) | 0.526 (0.430-0.629) | 0.457 (0.274-0.626) |
|  | SII | 0.572 (0.510-0.633) | 0.607 (0.507-0.706) | 0.573 (0.473-0.666) | 0.481 (0.300-0.646) |
|  | SIRI | 0.556 (0.494-0.619) | 0.556 (0.446-0.661) | 0.548 (0.450-0.645) | 0.451 (0.272-0.632) |
|  | AISI | 0.575 (0.512-0.637) | 0.590 (0.490-0.701) | 0.575 (0.477-0.668) | 0.457 (0.283-0.620) |
| Internal validation set | ML Score | 0.729 (0.639-0.804) | 0.746 (0.560-0.892) | 0.725 (0.582-0.853) | 0.755 (0.604-0.884) |
|  | Conventional Cox | 0.631 (0.528-0.737) | 0.588 (0.368-0.789) | 0.638 (0.486-0.778) | 0.643 (0.466-0.799) |
|  | NLR | 0.653 (0.555-0.744) | 0.735 (0.590-0.862) | 0.626 (0.482-0.761) | 0.561 (0.366-0.742) |
|  | SII | 0.660 (0.553-0.751) | 0.727 (0.529-0.882) | 0.675 (0.544-0.797) | 0.516 (0.326-0.687) |
|  | SIRI | 0.676 (0.574-0.768) | 0.739 (0.593-0.876) | 0.631 (0.498-0.767) | 0.714 (0.541-0.873) |
|  | AISI | 0.673 (0.567-0.764) | 0.723 (0.538-0.884) | 0.658 (0.527-0.783) | 0.655 (0.479-0.819) |
| External validation set 1 | ML Score | 0.718 (0.625-0.809) | 0.776 (0.555-0.908) | 0.699 (0.573-0.826) | 0.662 (0.350-0.932) |
|  | Conventional Cox | 0.679 (0.574-0.779) | 0.788 (0.669-0.879) | 0.586 (0.402-0.764) | 0.559 (0.286-0.832) |
|  | NLR | 0.615 (0.507-0.724) | 0.572 (0.370-0.762) | 0.634 (0.440-0.796) | 0.792 (0.587-0.942) |
|  | SII | 0.663 (0.558-0.763) | 0.664 (0.436-0.843) | 0.676 (0.518-0.818) | 0.790 (0.551-0.960) |
|  | SIRI | 0.690 (0.594-0.782) | 0.707 (0.577-0.836) | 0.725 (0.576-0.844) | 0.837 (0.659-0.963) |
|  | AISI | 0.707 (0.615-0.795) | 0.769 (0.656-0.883) | 0.718 (0.587-0.841) | 0.816 (0.579-0.981) |
| External validation set 2 | ML Score | 0.705 (0.626-0.786) | 0.711 (0.561-0.843) | 0.738 (0.613-0.842) | 0.771 (0.559-0.936) |
|  | Conventional Cox | 0.686 (0.582-0.781) | 0.737 (0.594-0.864) | 0.737 (0.607-0.853) | 0.767 (0.560-0.927) |
|  | NLR | 0.583 (0.482-0.682) | 0.666 (0.482-0.873) | 0.581 (0.442-0.719) | 0.675 (0.431-0.861) |
|  | SII | 0.665 (0.564-0.759) | 0.791 (0.628-0.936) | 0.633 (0.497-0.763) | 0.717 (0.481-0.903) |
|  | SIRI | 0.664 (0.561-0.760) | 0.820 (0.681-0.930) | 0.682 (0.549-0.808) | 0.627 (0.400-0.831) |
|  | AISI | 0.716 (0.609-0.814) | 0.863 (0.699-0.973) | 0.696 (0.559-0.819) | 0.698 (0.466-0.886) |

Abbreviations: ML, machine learning; NLR, neutrophil-to-lymphocyte ratio; SII, systemic immune-inflammation index; SIRI, systemic inflammation response index; AISI, aggregate index of systemic inflammation; AUC, area under the curve; CI, confidence interval.
